# Paradoxical Effects of a Cytokine and an Anticonvulsant Strengthen the Epigenetic/Enzymatic Avenue for Autism Research

**DOI:** 10.3389/fncel.2020.585395

**Published:** 2020-11-11

**Authors:** D. G. Béroule

**Affiliations:** ^1^CNRS, Bat.508, Faculté des Sciences d’Orsay, BP 133, Orsay, France; ^2^CRIIGEN, Paris, France

**Keywords:** autism spectrum disorders, brain enzymes, epigenetics, cytokines, short-chain fatty acids, gut microbiome, MAOA, guided propagation networks

## Abstract

Maternal exposure to the *valproate* short-chain fatty acid (SCFA) during pregnancy is known to possibly induce autism spectrum disorders (ASDs) in the offspring. By contrast, case studies have evidenced positive outcomes of this anticonvulsant drug in children with severe autism. Interestingly, the same paradoxical pattern applies to the *IL-17a inflammatory cytokine* involved in the immune system regulation. Such joint apparent contradictions can be overcome by pointing out that, among their respective signaling pathways, valproate and IL-17a share an enhancement of the “*type A monoamine oxidase*” (MAOA) enzyme carried by the X chromosome. In the *Guided Propagation* (GP) model of autism, such enzymatic rise triggers a prenatal epigenetic downregulation, which, without possible *X-inactivation*, and when coinciding with genetic expression variants of other brain enzymes, results in the delayed onset of autistic symptoms. The underlying imbalance of synaptic monoamines, *serotonin* in the first place, would reflect a mismatch between the environment to which the brain metabolism was prepared during gestation and the postnatal actual surroundings. Following a prenatal exposure to molecules that significantly elicit the MAOA gene expression, a daily treatment with the same metabolic impact would tend to recreate the fetal environment and contribute to rebalance monoamines, thus allowing proper neural circuits to gradually develop, provided behavioral re-education. Given the multifaceted other players than MAOA that are involved in the regulation of serotonin levels, potential compensatory effects are surveyed, which may underlie the autism heterogeneity. This explanatory framework opens up prospects regarding autism prevention and treatment, strikingly in line with current advances along the gut microbiome–brain axis.

## Introduction

**Figure 1:**
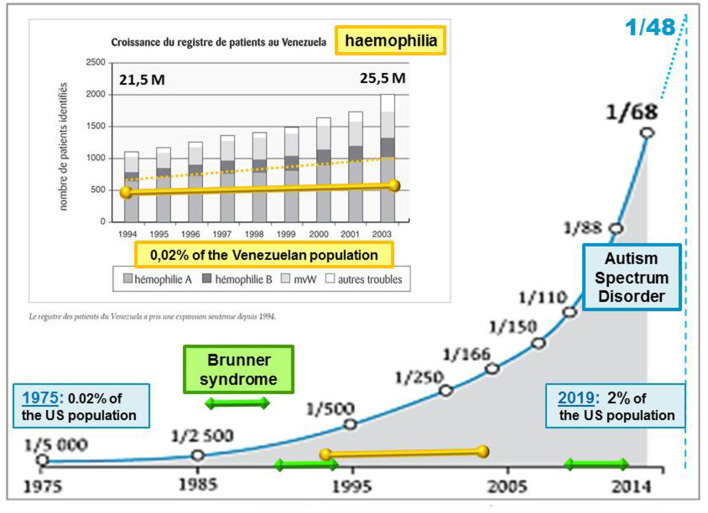
Evolution of the autism rate (blue asymptotic curve, from Demeneix, 2014) compared with genetic diseases: hemophilia (yellow line superimposed on a graph (excerpt from Evatt, 2010) and Brunner syndrome (BS; displayed by green line segments). Intellectual disabilities associated with BS, as observed in only two families around 1993 and 2013 (Piton et al., 2014), prevent transgenerational transmission, contrary to hemophilia, which can be passed on by healthy carriers from one generation to the next. By contrast with BS, the rate of hemophilia follows at least a slight progression, as reflected by a Venezuelan survey of affected patients over a decade (Evatt, 2010). The asymptotic increase of autism spectrum disorder (ASD) cases is proposed here to result from environmental factors inducing the silent inheritance of an epigenetic mark. The latter would remain hidden until genetic features are met across human generations. Accordingly, the ASD rates currently released would only account for cases of “overt autism”, irrespective of “healthy carriers” of the epigenetic mark at issue.

Living organisms do not passively undergo inputs from their biological environment. Whether air particle, radiation, food, or drug, any ambient stimulus generates different net effects depending on both its strength and context of intrusion into the human body. Contradictory global effects can even be initiated by the same input and can raise a paradox deserving examination for possibly gaining insights into the biological mechanisms at work. Although not considered as fully “environmental” (Modabbernia et al., 2017), *autism spectrum disorder* (ASD) cases have followed an asymptotic increase in number over the last 50 years (Demeneix, 2014), too rapidly to argue a clear genetic cause like in *hemophilia* (Evatt, 2010; Figure 1). Rather than being attributable to a significant genetic mutation, such as *Fragile X*, *Brunner* (Piton et al., 2014), and *Rett* syndromes among close conditions, ASD is usually described by several symptoms of variable severities. Communication deficits are likely to result from a lack of emotional guiding and control of perception, worsened in most severe cases by irrelevant repetitions of a few acts (*stereotypy*). A high comorbidity evokes a condition of the systemic type, including poor sleep, digestive problems, and epileptic signs.

The survey reported here has been motivated by the identification of an intriguing phenomenon relating recent trials of molecules with potential impact on autism. As a matter of fact, paradoxical data were issued by distinct scientific experiments whose respective molecular targets [i.e., *IL-17a cytokine* and *valproate* (VPA) anticonvulsant] were found to alleviate autistic symptoms (Yim et al., 2017; Aliyev and Aliyev, 2018), albeit previously seen as “at risk” if absorbed by the embryonic brain (Schneider and Przewłocki, 2005; Christensen et al., 2013; Wong and Hoeffer, 2018). In other words, separate studies suggest that some molecules can be implicated in the etiology of ASD when interfering with gestation, while providing potential remedies against overt autism. This issue requires clarification, notably in order to avoid promising treatments being shadowed by the knowledge of their adverse effects on pregnancy. More fundamentally, resolving this paradox may shed new light on the genesis of autism, as well as on its prevention and potential treatments.

## Context-Dependent Effects of Drugs

Life is communication. Indeed, living beings are driven by complex networks of signal transmission and control, within and between their constituent cells. More precisely, biological interactions between the cell and its microenvironment are performed through “cell signaling”, thanks to small proteins named *cytokines*, functionally close to hormones. At the macro-environmental level, drugs contain chemicals aimed at favorably interfering with the signaling cascades that interweave within the human body. Not surprisingly then, any single drug is likely to activate many such pathways, including the subnetworks responsible for protecting the host organism against pathogens: the *immune system*. Moreover, the expression of genes—orchestrated by *epigenetics*—can be disrupted by pharmaceutical formulations (Csoka and Szyf, 2009). In any case, the impact of a given drug depends on possible coincident stimuli (e.g., *polypharmacy*), plus the ongoing business of the host organism, as well as its background (epi)genetic traits. For example, the efficacy of a subclass of anti-hypertension agents (*beta-blockers*) undergoes variations throughout the day, coincidently with systems that control the blood pressure (Morgan and Anderson, 2003). Of note here, pregnancy constitutes a unique period during which most drugs must be either avoided or prescribed with caution, because critical periods of fetal development especially enhance drug side effects involving epigenetic and immune systems. With respect to ASD, exposure to the following molecules, either “during” or “after” pregnancy, may either “induce” or “reduce” symptoms.

1. *Cytokine IL-17a* is a small protein that contributes to cell signaling; it belongs to the *interleukin* family on which the immune system relies for regulating the maturation and responsiveness of cellular populations. IL-17a triggers signals aimed at recruiting white blood cells involved in immunity, while promoting the expression of anti-microbial *peptides*. It is important to add here that IL-17a is known to enhance the action of *IL-13*, an anti-inflammatory interleukin (Hall et al., 2017).
2. VPA is a synthetic short-chain fatty acid (SCFA), chemically similar to a molecule found in valerian. Among several medical uses, VPA is primarily an antiepileptic drug, which, in the early century, turned out to be deleterious for the offspring when prescribed across pregnancy.

## Two Molecules in Question

Temporary improvements have been reported in autistic individuals who experienced fever (Yim et al., 2017; Grzadzinski et al., 2018) or followed an antiepileptic treatment based on VPA (Hollander et al., 2001; Aliyev and Aliyev, 2018; Béroule, 2019). As introduced above, such reduction of symptoms is surprising because both medical conditions can also produce adverse effects during pregnancy (Patterson, 2011; Meador et al., 2013). At first sight, the same initial event (e.g., infection or drug) may therefore result in opposite final effects depending on the context, either embryonic or “postnatal with ASD.”

### Interleukin-17a

A large-scale study of children born in Denmark between 1980 and 2005 found that severe viral infections occurring within the first trimester of gestation increased by a factor of three the risk for autism in the offspring (Atladóttir et al., 2010). A molecular basis was given to account for this epidemiological study, namely, the elevation of cytokines associated with maternal immune response (Patterson, 2011). Among the set of pro-inflammatory actors that play their part in fighting infection, a significant contribution comes from interleukin-17a signaling. In pregnancy with maternal inflammatory condition, the activation of IL-17a pathways in the placenta may indeed predispose to the postnatal onset of ASD-like deficits (Choi et al., 2016; Wong and Hoeffer, 2018). Specifically, in pregnant mice, intestinal *Th17* cells, which produce IL-17a and are released by the human bacteria, are more likely to cause inflammation-associated abnormalities in the offspring (Kim et al., 2017). Other investigations evidenced that cytokine IL-17 participates in several neurological diseases, notably through the disruption of the *blood–brain barrier* (BBB) as well as a direct effect on brain cells (Cipollini et al., 2019). Unexpectedly, then, episodes of fever have been shown to temporarily alleviate aberrant behaviors in some autistic children (Curran et al., 2007). A molecular–behavioral link is now proposed between the release of IL-17a and the temporary reduction of impairments in animal models of autism (Reed et al., 2020). According to the authors of the relevant study, maternal exposure to this cytokine would impact a brain region (*S1DZ*) located in the somatosensory cortex, by getting bound to receptors and thus reducing neural activity in this cortical area (Yim et al., 2017). Autistic symptoms would result from the disturbed development of S1DZ during gestation. Near inhibition of the same region through IL-17a release would then restrict its defective functioning. However, it remains unclear how reducing the activity in this specialized brain area could either generate long-term wide-spectrum deficits or temporarily inhibit them, afterwards.

### Valproate

In 2006, building on previous work concerning the effectiveness of VPA against mood lability and irritability (Hollander et al., 2001), a 2-month, double-blind, placebo-controlled trial tested its benefits on stereotypy in ASD (Hollander et al., 2010). Based upon these preliminary results and the link found between isolated epileptiform discharges and deficits in attention, language, and behavior (Spence and Schneider, 2009), a clinical trial was planned in 2014 for investigating several medical outcomes of VPA. Because not enough subjects could satisfy the enrolment criteria, including the presence of epileptiform discharges, this study was withdrawn. No clinical trial was carried out until the end of the decade, preventing VPA from being assessed as a potential treatment against autistic symptoms. Incidentally, the US Food and Drug Administration (FDA) had advised health professionals mid-2013 that anti-seizure drugs based on VPA were contraindicated for pregnant women. This alert was supported at that time by a study showing that children exposed to VPA while their mothers were pregnant had significantly lower IQs at age 6 than children exposed to other antiepileptic drugs (Patterson, 2011). Consequently, the pregnancy status of VPA had then been shifted from a “possible use despite potential risks” to the “X” category warning that the risk of use clearly outweighs any possible benefit. In France, a 2015 administrative report stated that existing medical alerts had not accurately informed about known risks for pregnant women (Chastel et al., 2015). As a matter of fact, up to 40% of women exposed to VPA during pregnancy had given birth to children with intellectual disabilities and autism. Meanwhile, on the research front, a computational model of autism (Béroule, 2018) predicted that besides its classical anticonvulsant properties, VPA could serve as an autism-modifying drug for its capacity to promote the *type A monoamine oxidase* (MAOA) genetic expression (Gupta et al., 2015). In 2015–2016, a preliminary case study concerned the daily intake of a VPA-based anticonvulsant, monitored over 12 months in an 11-year-old child with severe autism and epileptic signs. Gradual improvements arose across a broad autism spectrum, some of them showing up only 9 months after the trial beginning, and sometimes disturbed by bursts of overactivity. In order to remedy the latter, *methylphenidate* could complete VPA at the pharmacological level, without mutual interference (Béroule, 2019). This favorable outcome has been corroborated by a double-blind placebo-controlled trial of the same VPA-based drug, involving 100 children and using a global rating scale of ASD; 80% of the treated subjects showed significant global improvement, compared with 12% in subjects having received a placebo (Aliyev and Aliyev, 2018). The researchers who conducted this clinical study eventually gave neurobiological interpretations that are often put forward to explain autism, relating the amplitude of sodium-dependent action potentials, as well as the inhibitory GABA neurotransmitters. However, neither heterogeneity of ASD nor paradoxical effects of specific molecules like VPA can currently be enlightened by the only global excitatory/inhibitory imbalance of brain neural circuits (Uzunova et al., 2015).

## Persistent Adaptation to the Fetal Environment

At this stage, the paradox raised by inconsistent effects of two molecules can be addressed through the following couple of questions.

1. What kinds of physiological events, caused by the same input, could either (during gestation) underlie the development of an ASD case or (in the autistic child) facilitate the onset of proper behavior?
2. Are there common signaling pathways at the intersection of those activated by the molecules under focus here? Accordingly, if all autism inducers shared a chemical cascade leading to ASD onset, any of them could form the basis of a potential remedy, regardless of the condition actual initiators.

In attempting to answer the above questions, the human broad adaptability can first be reminded and associated with the trend to keep track of environmental stimuli in the long run. Even early molecular memory acquired in the fetal life may thus modulate the impact of postnatal inputs. By contrast, the seemingly sudden onset of autistic signs in the infancy is still believed to readily result from early medications, especially vaccination, despite epidemiological studies based on large populations evidencing no such causal relationship (Hviid et al., 2019). The occurrence of symptoms before the age of 3 can less directly be linked to prenatal adverse events memorized in some way and silently integrated into the developmental script of the infant until a revealing situation. As a matter of fact, not so many biological systems (e.g., neural and immune) are capable of reliably implementing both “print” and “reading” memory functions in the long run. Of noticeable exception is that the DNA gene pool sustainably crosses generations, although experiencing rare mutations. Additionally, the embryonic genetic programming of stem cells—through epigenetics—propagates unaltered identities along cell lines (e.g., *X chromosome inactivation*; Pinheiro and Heard, 2017) and, under certain conditions, over human generations without genetic mutations: among pathologies of epigenetic origin, defects are still reported in the third generation after the grandmother was treated with *diethylstilbestrol* during a pregnancy carried in the third quarter of the 20th century (Gore et al., 2015). Beyond this transgenerational pathology, it seems that critical periods of embryonic brain development (Kim et al., 2011) exhibit enhanced susceptibility to the environment. Quite possibly, an early “snapshot” of this environment may be taken as reference for the baseline expression of “regulating genes” being fixed once and for all (e.g., MAOA enzyme). In particular, the synaptic concentration of neurotransmitters is likely adapted to the maternal health and feeding resources conveyed by the composition of the mother’s blood. With the latter being passed through both placenta and BBB to the fetus neural system, the neurotransmitters’ metabolism could be tuned so as to account for environmental signals. But this partial information may not actually reflect the chemical surroundings that the newborn will find after the end of gestation, when breastfeeding is over. In case of significant gap between (maternal) signals received in the womb and (autonomously) after birth, weaning may resemble a sort of withdrawal (a well-known example is the newborn of a smoking mother, whose brain does not receive any more psychotropic molecules). This environmental mismatch would be harmless if the early genetic programming could be refined according to the postnatal context, encompassing the erasure of obsolete epigenetic marks. Otherwise, a closer fit of prenatal and postnatal situations may however be reached by bringing the child chemical environment closer to influential events experienced in the mother’s womb. Although unusual, this view is consistent with the paradoxical situations addressed here. Environmental feature(s) to the metabolic effects of which the fetal brain got permanently adapted during a critical stage of gestation would thus favorably be brought into play. But apart from a few identified factors such as maternal infection, VPA-based treatment, or polluted surroundings (von Ehrenstein et al., 2019), the origin of a given case of autism remains often uncertain. Furthermore, not every environmental item, such as a pesticide, can form the basis of a safe treatment. These difficulties bring us back to the second question concerning the possibility that, although respectively generating various physiological outcomes, all potential initiators of an autistic case could share a subset of signaling pathways. Interestingly, this would allow molecules that are medically safe to partly reinstate the “lost environment” for which neuronal stem cells had been programmed into the womb.

## Shared Brain Enzymatic Pathways

**Figure 2:**
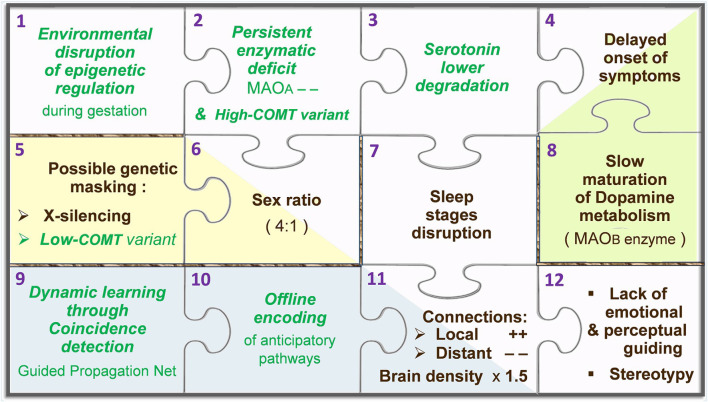
Puzzle of autism, according to the Guided Propagation (GP) model. Pieces containing a green label in italics represent hypotheses, while labels in black indicate actual facts. The main stream of pieces goes from the epigenetic regulation hypothesis (top-left piece, no. 1) towards core symptoms (bottom-right piece, no. 12). Two-tone pieces (nos. 4, 6, and 11) stand for facts that appear downstream of two pieces coding for a hypothesis and another fact: in this theoretical framework, the disrupted architecture of sleep (no. 7) is caused by a poor degradation of serotonin (no. 3), which leads to aberrant neural paths (no. 11) that are built “offline” in the anticipation mode normally only relevant “online” (no. 10). For further details, see Béroule (2018).

Among neurotransmitters, *monoamines* are modulating agents involved in the control of behavior and learning. The ongoing synaptic concentration of monoaminergic neurotransmitters is therefore central in the brain function, relying on a subtle balance to be preserved between their production/*synthesis*, *recycling*, and degradation/*catabolism*. Reabsorbed through *reuptake* into the afferent synapse, monoamines can either be used again (recycled) or be degraded in the presynaptic neuron by MAOA, while another enzyme, named *catechol-O-methyl transferase* (COMT), works in the postsynaptic neuron. The potential for regulated trafficking between relevant systems relies on early epigenetic setup, as well as feedback loops to properly adjust metabolic parameters. Provided an optimal range of synaptic levels for a given neurotransmitter, the inner promotion of its catabolism may for instance be set “down” and its synthesis be boosted in case of a too-low concentration. Such out-of-range output may be due to an accidental input involving for instance MAOA. The A type of MAO, carried by the X chromosome and known as a vital regulator of embryonic brain development (Wang et al., 2011), is capable of degrading at least three key monoamines, namely, *dopamine* (DA), *serotonin* (5-HT), and *norepinephrine* (NE), whereas type B monoamine oxidase (MAOB) and COMT cannot catabolize 5-HT. On first analysis, this discrepancy is likely to elicit unbalanced synaptic concentrations between 5-HT and other monoamines. However, as long as MAOA remains the main player to carry on the degradation of all monoamines, their levels consequently decrease at the same rate. In the *Guided Propagation* (GP) model of autism, monoaminergic equilibrium tends to continue if COMT is poorly expressed, if X-inactivation occurs (in women), and until MAOB is fully operational (around 2 years after birth). At the computational level, the higher the local parameter *Da* that codes for DA, the more controlled the response of *elementary processing units* (EPUs) despite possible imbalance of other monoamine-like parameters; this occurs when the “*Da* concentration” is decreased, which in the real world accompanies an increase of MAOB activity. A theoretical scenario therefore begins with an accidental stimulation of MAOA during gestation, eliciting a long-term epigenetic regulation of the same enzyme in the embryonic neurons. This epigenetic mark would become deleterious when meeting high-functioning COMT promoter, and without protection by X-inactivation (because all X chromosomes carry the downregulated MAOA). The relatively poor degradation of 5-HT assumed here would only initiate visible effects in the early childhood, when the third metabolizer (MAOB) is mature enough to supply the baseline catabolism of DA. This enzymatic reading provides an explanation for the regressive feature of ASD, while the X-inactivation and COMT polymorphism (low to high expressions) can together account for the male prevalence (Béroule, 2018, 2019). Consequent structural deficits are represented by a GP network in which the local *5ht* parameters decrease slower than *Ne* across offline encoding, inducing either aberrant or lacking memory pathways (Béroule, 2016). The higher the *5ht*/*Ne* ratio, the wider the misconnections, namely, the chaining of meaningless sensorimotor patterns, magnified convergence of inner stimuli towards EPUs inducing their overactivity, and missing links between channels that normally cooperate in sensorimotor tasks. For the sake of simplicity, the way the above events are represented in the GP model can be displayed by an assembly of puzzle pieces (Figure 2).

Given that the downregulation of MAOA is being proposed as key crossover point between prenatal signaling and a spectrum of postnatal symptoms, the second question to be addressed is about the generic nature of this central feature. Ultimately, is IL-17a able to affect MAOA just as VPA?

As stated above, VPA is already known to stimulate the MAOA gene promotion, through select signal pathways (Gupta et al., 2015). Of note, several “medium-chain” fatty acids and SCFAs share chemical and structural properties with VPA, making them potential MAOA inducers; they are named *pelargonic/nonanoic*, *decenoic*, *propionic*, *butyric*, and *valeric* from which VPA is made. Now, with regard to the IL-17 cytokine, no direct action was reported towards MAOA. But other molecules activated by IL-17 could promote MAOA and therefore be responsible for ASD cases through a mathematical *transitive relation*. Indeed, MAOA is one of the most strongly upregulated gene within cells activated by the IL-13 anti-inflammatory interleukin (Dhabal et al., 2018), which gives the following: (1) IL-13 → MAOA+. Not surprisingly then, elevated gestational IL-13 associated with maternal inflammatory immune response and maternal–fetal cytokine signaling increases the risk for the offspring to develop abnormalities, namely, ASD, hyperactivity, and inattention (Thürmann et al., 2019). Importantly here, the genetic expression of IL-13 is enhanced by the pro-inflammatory IL-17, i.e., (2) IL-17 → IL-13, provided that their signaling pathways are present in the same cell (Hall et al., 2017). Indeed, IL-13 can be produced inside the brain, where its receptors have been evidenced (Mori et al., 2016); for its part, the IL-17 generated outside the brain is conveyed by the blood, can get across the fetal BBB, and then reach receptors (IL-17R) found in neural cells (Luo et al., 2019), together with IL-13 receptors. Taken together, (1) and (2) lead to IL-17 → MAOA+.

To sum up, the paradoxes that motivated this study only appear from a distant point of view. A close-range focus shows an immediate common effect of molecules at issue, regardless of context, namely, an increased promotion of the MAOA enzyme. In the GP model, second-order effects differ according to the context of exposure:

1. Blank situation, critical period of neurulation: The monoamine metabolism is durably adapted to the current fetal environment through MAOA downregulation (MAOA–in Figure 2). When the transport of causal molecule(s) by maternal blood/milk is discontinued, no later than the end of gestation/breastfeeding, the MAOA setting remains.
2. Then, the occurrence of MAOA inducers is expected by the epigenetic memory to comply with its prenatal regulation. A metabolic imbalance may otherwise result from the high-expression variant of COMT and be enhanced by matured MAOB.
3. In case of epigenetic masking (i.e., X-inactivation in women) or genetic variant (low/medium COMT), the MAOA downregulation is silently transferred to the next offspring where it may occur in a different genetic context leading to overt autism (case 2 above).

**Figure 3:**
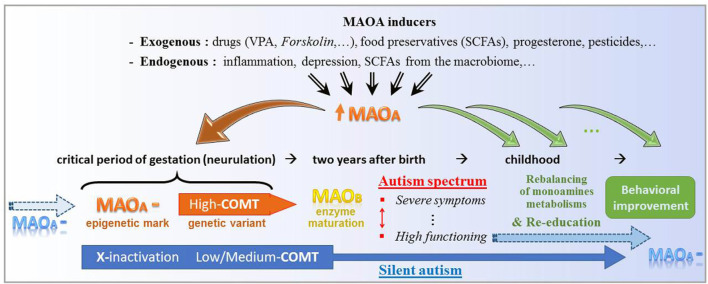
Theoretical key functions of the molecular stimulation of type A monoamine oxidase (MAOA) in either initiation or alleviation of autism. At the top, several non-genetic factors share the property to significantly promote the expression of the MAOA gene. In the early gestation, an epigenetic downregulation of MAOA (MAOA—), which notably combines here with genetic variants of the catechol-*O*-methyl transferase (COMT) enzyme, may be inherited (blue arrow at the left-hand side) and/or favored/caused by MAOA enhancers (brown down arrow to the left). At the right-hand side, the series of smaller green arrows stand for a daily intake of MAOA inducer, which may bridge the gap between gestational and postnatal respective environments by reducing the metabolic imbalance of monoamines and consequently support behavioral re-education. At the bottom, a genetic variant (low-to-medium expression of COMT) and the classical epigenetic X-inactivation may hide the “MAOA—” mark, which is however silently carried towards the next human generation.

According to this possible etiology of ASD (Figure 3), only the erasure of specific epigenetic traits supporting the MAOA downregulation could permanently reverse the brain enzymatic imbalance at issue. Disease-modifying treatments based on MAOA stimulation nevertheless represent an alternative thanks to neurogenesis and targeted neural migration. Favored by an educational program, the gradual growth of proper neural structures is assumed to support the enrichment of social conduct, at the price of a sustained supply of drugs aimed at approaching the embryonic microenvironment. The sooner the treatment initiation, the better its expected outcomes. However, while shown to be effective against both epileptic signs and some ASD symptoms, VPA-based drugs can be toxic to detoxification organs, most commonly below 6 years of age (Star et al., 2014), encompassing the earlier time at which behavioral deficits start arising. Alternative treatments remain to be established with the aim of being safe at an early age. Accordingly, one should care about recent strides in the understanding of the *gut microbiome* function.

## The Microbiome Resources

The *enteric nervous system* (ENS), also referred to as the “second brain,” notably allows some autonomy in the management of digestion, however mediated by the same chemical mediators as those at work in the “first” brain. It logically follows that a systemic metabolic imbalance of monoamines is likely to affect the neural cells of both nervous systems. In the close neighborhood of trillions of microbes, some of which induce inflammation and produce SCFAs, the ENS appears to be better equipped than the brain to deal with a metabolic dysregulation. Molecules elicited by the gut microbiota mostly serve local functions before being degraded in the liver, but those which can reach the blood circulation (*via* the *distal colon* bypass) and get through the BBB are eventually able to spread their metabolic influence across the brain. In particular, regulatory outcomes primarily aimed at the second brain may benefit the first one. Besides a common lowered diversity of the autistic children microbiome, a trend seems to emerge from existing data, reminiscent of the alternative ASD inducer/remedy represented here by cytokines and SCFAs. In absence of medical intervention involving antibiotics, the microbiota would develop along the inflammatory arm and its associated digestive troubles: any shift towards a gut pro-inflammatory state can in turn trigger the activation of neuro-inflammatory responses in the brain (Golubeva et al., 2017), with potential improvement of ASD. A relatively low concentration of SCFAs can then be observed in the plasma of autistic children (El-Ansary et al., 2011). When reset by a strong antibiotic, short diet, and replaced by typical germs, following a *microbiota transfer therapy*, the SCFA by-products arm can be privileged in a long-lasting way (Kang et al., 2017). Data obtained *in vitro* by using a neuron-like embryonic cell-line notably demonstrate the regulation exerted by propionic acid (PPA) and butyric acid (BA; i.e., *propionate* and *butyrate*) over the expression of neural genes (Nankova et al., 2014). Among a large set of genes tested for their link with ASD, the MAOA expression is doubled by each of these acids, while COMT is decreased by a factor of 2.7 (which means a total of 7.3 if assuming a multiplicative combination of the two acids). Whereas both genetic modulations alleviate the harmful setting pointed out by the GP model (MAOA–and high COMT), therefore suggesting a corrective action of the microbiome, the latter is rather usually believed to exacerbate ASD symptoms (Goines and Ashwood, 2013; Slattery et al., 2016). Even at odds with the theory presented here, immediate behavioral worsening has been proposed to result from exposure to SCFA. Rats whose brain directly receives PPA show epileptiform activity within the 30 min of infusion, accompanied by a variety of abnormal responses (retropulsion, hyperextension, turning, and dystonia) considered as “bearing some resemblance” to bouts of irrelevant actions found in autism (MacFabe et al., 2007). Bacteria that produce SCFAs and elicit inflammation can thus be viewed as unwelcome guests in the gut of subjects with ASD. This position is now challenged by a study in which significant improvements of 18 children with ASD occurred together with the colonization of the gut by antibiotic-resistant and SCFA-producing bacteria (*Bifidobacterium*, *Prevotella*, and *Desulfovibrio*). Persistency of this favorable effect has been confirmed in the same subjects, 2 years after their initial enrollment. Both *Prevotella* and *Desulfovibrio* were on average more abundant in ASD subjects following treatment than in the donor samples: *Prevotella* was 712 times higher after 6 weeks and maintained an 84-times increase after 2 years (Kang et al., 2019). Although Clostridia and *Bacteroides* are also reported as PPA producers in ASD, they share with *Sutterella* the joint occurrence of gastrointestinal disorders such as inflammatory bowel disease (Bezawada et al., 2020). An added plus in favor of the *Prevotella* prevalence is the production of up to three times more PPA than the *Bacteroides*-dominated microbiota associated with high-fat intake (Chen et al., 2017). If PPA was confirmed as effective against overt autism, evidence of its deleterious effects on gestation would provide further support to the present theory. Unexpectedly, such indication already exists. As a matter of fact, *propionic acidemia* is a genetic disease—obviously affecting early gestation—in which the accumulation of PPA elicits several metabolic disorders, including seizures, gastrointestinal disturbances, intellectual disability, and delayed development (Pena and Burton, 2012). In addition, a microbiome shift in maternal gut, leading to formation of PPA and BA by-products during the early stages of the fetus’ neural development, has been linked to ASD (Abdelli et al., 2019).

Through daily intake, VPA primarily protects against epilepsy. A preliminary trial led to its association with the methylphenidate psychostimulant for balancing the monoaminergic metabolism (Béroule, 2019). By comparison, the possible natural production of propionate and butyrate by the microbiome, following a process for initiating the relevant change (Kang et al., 2017), may be preserved by a high-fiber diet. Furthermore, PPA and BA would properly regulate MAOA and COMT, whereas VPA proved to require a complementary medication. Although this avenue still requires closer analyses and refinements, the gut microbiota are therefore likely to provide an alternative to the pharmacological approach of ASD. Regarding now the protection of gestation, SCFA gut by-products should be restricted, particularly when realizing that they would add to similar substances widely used as anti-mold/preservative ingredients in the food industry (Abdelli et al., 2019). Together with the immune system, the microbiome may consequently require follow-up when planning pregnancy, as part of a strategy aimed at limiting autism development (Paysour et al., 2019).

## Discussion: Allocation of Roles Among Networks of Players

### Facing the Autism Complexity

In 2020, a wide-angle approach to the wide spectrum of autism can be adopted (e.g., Marotta et al., 2020). Given the myriad of various factors that have been linked to ASD over the past decades, a shared responsibility is assumed today, however possibly modulated by emphasis on a selection of signaling pathways. At the molecular level, interdependent systems control the expression of numerous susceptibility genes constrained by their respective polymorphisms and rare mutations, and moreover submitted to environmental stimuli through epigenetics. A strict focus on any of these participants is thus challenged by the known involvement of many other players. With regard to neurotransmitters (Cetin et al., 2015), their metabolic breakdown by brain enzymes only concludes a series of contributions for them to be synthesized, released in the synaptic space, and either recycled or degraded in the presynaptic neuron thanks to *transporters*, towards several subtypes of receptors. All along repetitive rounds of this “neurotransmitter cycle”, any player disruption is likely to alter the functions of its partners. Conversely and more positively, several partners may respond by implementing compensatory mechanisms, hence possibly repairing/masking an original impairment. An irrelevant molecular activity can thus be elicited in signaling cascades by upstream previous alterations as well as feedbacks from downstream events, whereas the impact of a local deficit may be hidden by the genetic background and ongoing homeostatic regulations. This situation confirms that the actual picture is obviously more complicated than the representation displayed in Figure 3.

### Pivotal Role of Serotonin

Despite the complex crosstalk between numerous candidates likely to underlie the ASD heterogeneity, a leading role can be attributed to one monoamine for its recognized contribution to the brain development. Among neurotransmitters, 5-HT has truly become a main biomarker of autism (Benza and Chugani, 2015; Muller et al., 2016). Half a century ago, elevated whole blood 5-HT (*hyperserotonemia*) was the first molecular phenomenon found in a subset of ASD subjects, today associated with 25–30% of cases. The fact that hyperserotonemia is not detected in every autistic case is consistent with the correlation of this feature with ASD severity (Abdulamir et al., 2018) and also reveals individual variations in the biological inner management of disturbances impacting the 5-HT system. Along this line, it is worth knowing first that the level of 5-HT contained in blood *platelets* does not reflect its synaptic concentration. The opposite relationship rather applies, notably through the SERT transporter, which transfers 5-HT from the “extra-” to “intra-” cellular space of neurons, glial cells, and platelets. The 5-HT 2B receptor (5-HT_2B_R) signaling has recently been proposed to control this process thanks to a feedback loop that keeps synaptic 5-HT at tonic concentrations necessary for brain functions (Baudry et al., 2019). Consequently, a high synaptic level of 5-HT would promote its low uptake. 5-HT_2B_R, among other auto-receptors, thus participates in 5-HT tuning, which also relies on opposed contributions, namely, synthesis and degradation. The brain 5-HT precursor, named “type 2 tryptophan” (TPH2), is key in 5-HT synthesis and affects both 5-HT availability and synaptic levels in the opposite way as MAOA. These various agents form a team that allows a player irrelevant action to be alleviated by the overexpression/subexpression of other team members. Studies concerning either knock-out (KO) or overexpressed genes are informative of this partnership, aimed at keeping sufficient 5-HT concentrations in both presynaptic vesicles and synaptic cleft.

### Multiple-Source Serotonin Tuning

Targeted genetic modifications of the “5-HT team” members have provided animal models of autism to be compared with wild-type (WT) subjects (Muller et al., 2016). In SERT-KO mice, the aforementioned vesicles lack the “recycled 5-HT” input; 5-HT availability then depends on its upgraded synthesis and/or reduced breakdown. On the opposite side, in high-functioning SERT (*Ala56*) gain-of-function mutants, the related high-rate uptake favors the storage of 5-HT into vesicles at the expense of synaptic 5-HT signaling extent and duration. In both out-of-range situations, whether regulating factors could on the one hand optimize the availability of 5-HT packaged into vesicles and, on the other hand, could maintain tonic concentrations in the synapse represents a known conceptual problem for the mechanism of vesicular release (Blakely and Edwards, 2012). However, 5-HT-enhanced synthesis and reduced degradation can be suspected to: (a) better feed the pool of releasable 5-HT despite inactive reuptake (in SERT-KO mice); and (b) maintain the 5-HT synaptic level required for receptor activation despite hyperactive reuptake (in Ala56 mice). Consistent with this common solution, the 5-HT synthesis capacity has been found to increase gradually in autistic children, up to 1.5 times the adult normal value (Chugani et al., 1999), while a 30% deficit of MAOA activity has been identified in the cerebellum (for 70% of autistic children) and in the frontal cortex (for 55% of young subjects; Gu et al., 2017). Of note, such a depressed enzymatic activity cannot be witnessed by platelets since they only harbor MAOB. Amazingly enough, despite a significant *in vivo* increase in the rate of synaptic clearance in Ala56 vs. WT mice, no change was observed in brain tissue 5-HT levels (Veenstra-VanderWeele et al., 2012), which underpins the implication of compensatory processes. Moreover, the male full prevalence suggests the mediation of at least one protein whose gene is carried by the X chromosome (i.e., MAOA), besides sex-specific loci present in the SERT gene (Weiss et al., 2005). In addition, the surprising time delay of positive effects following SERT inhibition aimed at alleviating ASD symptoms in Ala56 mice (Robson et al., 2018) can be explained by the time-consuming neural migration necessary for new neural structures to be encoded and consolidated under well-regulated MAOA (Béroule, 2018).

After having pointed to metabolic implications of out-of-range SERT, let us start now from the downregulated MAOA hypothesis. In order to avoid a 5-HT synaptic overload caused by MAOA deficiency, possible counteractions include a stronger SERT gene expression, possibly less controlled by 5HT_2B_R. Indeed, the SERT gene promoter variants determine different levels of expression affecting 5-HT levels and social skills (Tanaka et al., 2018); a potent clearance of synapses can then be linked with the peripheral hyperserotonemia found in a subset of children with autism, likely carriers of the long allelic variants of the SERT gene promoter (Quinlan et al., 2020). On the opposite, low expressing short-allele of SERT in autistic children and SERT knock-out in mice are both associated with increased cortical volume (*macrocephaly*), counted as hallmark of severe autism. Either loss-of-function polymorphism or epigenetic downregulation of TPH2, the rate-limiting enzyme that allows *tryptophan* to be transformed into brain 5-HT, could modulate the biosynthesis of the latter (Chen and Miller, 2012). Consequently, a lower 5-HT concentration in the synapse would in turn generate the observed enhanced sensitivity of 5HT_1A_ and 5HT_2A_ postsynaptic receptors in mice mutants (Veenstra-VanderWeele et al., 2012). It eventually appears that the modulating agents mentioned above (TPH2 enzyme, SERT-based recycling system, and 5-HT receptors) are likely to counteract a chronic metabolic breakdown of synaptic 5-HT, while exerting a specific influence on ASD-nonspecific behaviors, such as the management of stress (Chen and Miller, 2012).

### Reviewed Position of Type A Monoamine Oxidase

The acute excess of MAOA, which is assumed to trigger an epigenetic reaction, may stand downstream a signaling cascade initiated by factors that stimulate the synaptic 5-HT at an early stage of the brain development. Among molecules holding this property, exposure of fetuses to *5-HT selective reuptake inhibitors* increases the risk of ASD (Harrington et al., 2014), unless the 5-HT_3_ receptor is knocked out (Engel et al., 2013). Within the GP framework, this receptor would be required to detect the 5-HT excess leading to an acute boost of MAOA, fostering then its persistent epigenetic downregulation. The scenario shown in Figure 3 is more fundamentally challenged by the fact that a MAOA deficit has not been evidenced in every case of autism. An imbalance activity between the transporter proteins in charge of monoamines (e.g., Low SERT) could then induce the 5-HT synaptic excess at issue, in which case MAOA would not represent a key factor in ASD etiology.

To conclude this discussion, variations of synaptic 5-HT induced by several interactive players may combine so as to cover the full ASD spectrum. Not systematically tested in connection with blood levels of 5-HT (Weiss et al., 2005) nor in SERT-oriented models of autism (Quinlan et al., 2020), MAOA and its enzymatic partners however deserve interest, in connection with other concerned agents, including SERT, 5-HT receptors, and TPH2 among a large family of neurotransmission regulators.

First, MAOA is the only enzyme to catabolize 5-HT, whose disruption is a prime ASD biomarker.

Second, the co-occurrence of a weak MAOA, high COMT, and mature MAOB can account for two ASD features that remained unexplained beforehand, namely, the onset of symptoms before 3 years of age and the sex ratio that partly results from the localization of MAOA on the X chromosome. Other characteristics of the condition can be simulated in a computational model that incorporates a representation of monoamine functions.

Third, among genetically modified rodents—whose neuromodulating system is comparable with the human one, MAOA-KO mice exhibit high correspondence to ASD core symptoms, which warrants its status of “animal model of autism.” In human, the very rare mutation of MAOA responsible for the BS has only been linked to autism 20 years after the former discovery of a family with serious behavioral troubles (Piton et al., 2014). At that time, according to the GP model of autism, ASD symptoms could have been masked by genetic variants such has low-COMT polymorphism, or immature MAOB enzyme, or DA overexpression. MAOA might have raised more awareness among researchers, otherwise.

Fourth but not false, the extinction of both 5-HT and NE across every sleep cycle cannot take full advantage of the online multiple regulations of 5-HT addressed above. If the balanced offset of these two monoamines turned out to hold a critical functional role, the disparate 5-HT levels observed online within the autism spectrum would eventually appear not so critical for the structural foundation of autism. Essentially, the GP theory puts forward a 5-HT “noise” that improperly remains into the synaptic cleft during sleep whatever the (non-null) baseline level of 5-HT at the beginning of every sleep cycle, therefore relatively irrespective of the online regulation exerted by the players introduced above.

## Conclusion: Natural Fatty Acids Under Focus

In our modern environment, possible enzymatic disruptions during gestation are substantiated by the finding of chemical factors that overexpress the MAOA enzyme and strikingly entail similar paradoxical effects. One purpose of the present article was to clarify this point, which included confronting a theory of autism to experimental data. An avenue for research now appears to widen, already suggesting medical approaches. In a few words, the goal would be to reinstate the enzymatic shift that caused a persistent epigenetic reaction through which the identity of neuron cell lines was irreversibly fixed in the early gestation (Figure 3). From a clinical point of view, this global prediction can be tested on the occasion of future trials concerning MAOA-inducing molecules. Relevant biomarkers have been proposed (Béroule, 2019), including the periodic control of sleep electroencephalogram (EEG), levels of monoamines, their metabolizers and metabolites, and, if possible, the family phenotyping of selected genes responsible for the management of monoamines.

Although not initially expected to be relevant, data on the gut microbiome turned out to strengthen the resolution presented in this article, while offering a potential medical resource. Feedbacks from the gut microbiota towards nervous systems can in fact be viewed as an attempt to reduce the enzymatic imbalance assumed here to underlie autism. As an aside, the continuous release of microbiota by-products remains challenging, since it may maintain the causal epigenetic traits in a sort of vicious cycle, a point to be raised in future works. Furthermore, studies focused on the microbiome enlarged the list of “at risk” industrial chemicals, the natural origin of which is often put forward by their marketer. Obviously, “natural” does not mean “safe.” It would be wise for women with a parenthood plan to avoid several products that contain SCFAs or medium-chain fatty acids close to VPA (e.g., food ingredient; Martinez-Mayorga et al., 2013), among other MAOA inducers such as *progesterone* (Kobayashi, 1980) and *forskolin*. Studied for inhibiting ASD progression in rat (Sidharth et al., 2020), forskolin is likely to enhance the MAOA promoter activity, at least in *Neuro2a* cells akin to neural stem cells, and particularly responsive to environmental factors (Gupta et al., 2015). It is also known as an effective weight-loss agent extracted from the Indian *coleus* plant, but its consumption is already not recommended for pregnant women (Godard et al., 2005). As an illustration of misleading advertisement, the *glyphosate* herbicide molecule has been replaced by the FDA-approved pelargonic acid, declared to occur naturally in many plants and animals. One may however bear in mind that pelargonic and valproic acids are close molecules and that permitted concentrations of the first one are comparable with the initial dosing of the second one for an anticonvulsant treatment, which is now forbidden during gestation (Béroule, 2019). Although potentially harmful during pregnancy, some of the molecules that stimulate the expression of MAOA could paradoxically form the basis of medications aimed at stopping the growth of aberrant neural structures, as soon as possible after the onset of autistic signs, hopefully giving conventional school a chance to be effective.

A shifted conception of autism emerges from the above organized set of data and hypotheses, as a side effect of systemic adaptation to environmental changes that, incidentally, could participate in the human evolution. Against this background, medical problems occur in case of critical mismatch between the after-birth surroundings of the brain and the gestational “snapshot” on which a persistent metabolic regulation of its monoamines is supposedly based. Accordingly, this instance of transgenerational epigenetics might one day reveal appropriate, if our environment became continuously overwhelmed by small/medium fatty acids in food, cosmetics, drugs, and pesticides, not mentioning inflammation associated with widespread viral dissemination.

## Data Availability Statement

Publicly available datasets were analyzed in this study. This data can be found here: https://perso.limsi.fr/domi/Movie-S1_DGB_nov16.mov.

## Author Contributions

The author confirms being the sole contributor of this work and has approved it for publication.

## Conflict of Interest

The author declares that the research was conducted in the absence of any commercial or financial relationships that could be construed as a potential conflict of interest.

## Abbreviations

ASD: autism spectrum disorder
BBB: blood–brain barrier
BS: Brunner syndrome
BA: butyric acid
COMT: catechol-*O*-methyl transferase
DA: dopamine
ENS: enteric nervous system
EPU: elementary processing unit
FDA: Food and Drug Administration
GP: Guided Propagation
SCFA: short-chain fatty acid
VPA: valproic acid
MAOA: type A monoamine oxidase
MAOB: type B monoamine oxidase
NE: norepinephrine
PPA: propionic acid
SERT: serotonin transporter
TPH2: type 2 tryptophan
5-HT: serotonin
5HT_2B_R: type 2B serotonin receptor
WT: wild type.
